# Arrest and non-fatal suicide attempts among men: analysis of survey data from the National Survey on Drug Use and Health

**DOI:** 10.1186/s12888-021-03544-0

**Published:** 2021-10-29

**Authors:** William C. Bryson, Jennifer Piel, Stephen M. Thielke

**Affiliations:** 1 Departtment of Psychiatry and Behavioral Sciences, University of Washington, Seattle, WA USA; 2 VA Puget Sound Health Care System, Outpatient Mental Health Service, Seattle, WA USA

**Keywords:** Suicide attempts, Law enforcement, Criminal justice, Social determinants, Public health, Epidemiology

## Abstract

**Background:**

Studies have found an association between recent arrest and suicide attempts, but the population-level significance of this link has not been reported. We estimated the population attributable risk percent (PAR%) of self-reported non-fatal suicide attempts based on recent arrest in a national sample of adult men.

**Methods:**

This study included men aged ≥18 who completed the 2008–2019 National Surveys on Drug Use and Health. The outcome measure was any non-fatal suicide attempts in the past year. The primary independent variable was any arrest in the past year. Major depression and substance use disorders were also included as independent variables for comparison. Descriptive statistics and multivariate logistic regression with postestimation marginal effects ascertained the PAR% of non-fatal suicide attempts for arrest, major depression, and substance use disorders, while controlling for sociodemographic covariates. All analyses applied survey weights. We disaggregated analyses by race/ethnicity.

**Results:**

In the sample of 220,261 men, arrest accounted for 8.9% (99% CI 5.1 to 12.6%, *p* < 0.001) of non-fatal suicide attempts, while major depression accounted for 40.3% (99% CI 35.0 to 45.1%. *p* < 0.001) and substance use disorders for 24.1% (99% CI 17.6 to 30.2%, *p* < 0.001). After disaggregating by race/ethnicity, arrest accounted for 9.5% (99% CI 4.5 to 14.3%, *p* < 0.001) of suicide attempts among Non-Hispanic White men and fell short of statistical significance for Non-Hispanic Black men (10.2, 99% CI − 3.0 to 21.6%, *p* = 0.043) and Hispanic men (8.1, 99% CI − 0.5 to 15.9%, *p* = 0.016).

**Conclusions:**

Arrest accounted for nearly one in eleven non-fatal suicide attempts in a national sample of American men, which is by extension about 50,000 suicide attempts per year. Results were similar for Non-Hispanic White, Non-Hispanic Black, and Hispanic men, although there were differences in prevalence of arrest and suicide attempts. Unlike major depression, arrest is an easily identifiable event, and the period after arrest might provide an opportunity to support mental health and coping.

## Background

American men had more than 37,000 suicide deaths and 550,000 nonfatal suicide attempts in 2018 (https://wonder.cdc.gov; https://afsp.org/suicide-statistics). Research and public health programs have attempted to identify preventable and modifiable risk factors for suicidal thoughts and behaviors, with a focus on mental health conditions [1, 2], substance use disorders [3, 4], and socioeconomic factors [5]. Most these studies, however, focused only on the adjusted rates of suicidality in groups with and without the risk factors, and did not account for their population prevalence. Without ascertaining prevalence, it is difficult to determine and address their public health significance.

Population attributable risk percent (PAR%) is a measure of association that considers both strength of association and prevalence of the risk factor in the population, which makes it relevant for the study of suicide at the population level [6]. PAR% can be interpreted as the percentage of suicide attempts attributable to a specific factor or set of factors, or, analogously, as the percentage of suicide attempts that would be prevented if the risk factor(s) were eliminated from the population.

Some prior population-based studies have calculated the PAR% for suicide attempts using a limited set of risk factors. Most have focused on psychiatric diagnoses, especially depression, typically finding the PAR% to be in the 25–35% range for recent major depressive disorder [7–10]. Additional studies focusing on substance use disorders reported PAR% that ranged from 5 to 15% in various samples [7, 11, 12]. A handful of other studies have focused on suicidality and various sociodemographic risk factors, including older age, childhood trauma, lower education, unemployment, and unmarried status [5, 13]. Although some of these cannot be prevented or readily modified, the results still provide useful information about how large-scale social and policy changes may influence population-level suicide rates.

Involvement in the criminal legal system is an established modifiable risk factor for suicide. Most studies at individual and population levels have focused on pre-trial detainment and community reentry following release from incarceration as periods of exceptionally high suicide risk. Pre-trial detainment, for example, accounts for 75% of all jail suicides [14]. Those awaiting trial are six times more likely to die by suicide than people imprisoned after being sentenced [15]. On the other end of incarceration, rates of suicide following release from prison are 3–18 times higher than the general population in various samples [16–18]. These data suggest that transitions into and out of the criminal legal system are unique and critical windows from a suicide risk and prevention standpoint. Looking furthest upstream, then, criminal arrest is the entry point into the criminal legal system from which these downstream risks propagate. Preventing arrest, or even lessening its stress and trauma, could mitigate the substantial downstream risks of suicide in the criminal legal system. Recent studies have begun to show that arrest may be more consequential than further downstream aspects of criminal legal involvement for mental health symptoms [19] and suicide attempts [20].

Despite the recent interest in criminal arrest as a preventable suicide risk factor, it has not been studied from a population perspective. A small but growing body of work has consistently associated arrest with suicide attempts, and authors have theorized a stress-diathesis causal mechanism for the association: the stress and trauma of arrest and associated criminal legal involvement may push individuals already at risk for suicide over the edge [20–24]. Another hypothesis that has been proposed to explain the association between arrest and suicide relates to the criminalization of mental illness, wherein socioeconomic hardships and disorganized, impulsive, or agitated behavior associated with untreated mental illness may increase the risks of both arrest and suicidality [25, 26].

While arrest is subject to the same PAR% limitations as other socioeconomic risk factors, there are important reasons to study it. First, arrest is relatively common, with over 10 million arrests every year (https://fbi.gov/services/cjis/ucr/), suggesting that even a relatively modest causal association with suicide could translate into a substantial number of attempts and completions. Second, the prevalence of arrests in the U.S. has changed in significant ways over recent decades, reaching peaks of 14–15 million per year at the height of mass incarceration in the 1990s and 2000s, and falling since then as concerns about police brutality have risen, especially towards African American men [27]. Professional organizations, including the American Medical Association, the American Psychiatric Association, and the National Association of County & City Health Officials have released policy statements condemning antiblack racism, its manifestation as police violence, and the public health consequences, urging action to counteract resulting health disparities [28–31]. The full public health consequences of arrest, including suicide attempts among arrestees, must be considered to make informed policy decisions.

In order to ascertain the association between arrest and self-harm, we calculated the PAR% of non-fatal suicide attempts for arrest, major depression, and substance use disorders among a large national sample of men. Major depression and substance use disorders were included primarily for comparison, as they are established risk factors for suicide. We focus on women in a separate but related study. In light of prior research on the disproportionate impact of the criminal legal system on the mental health of African American men [31], we conducted separate analyses by race/ethnicity, and compared results across them.

## Materials and methods

### Participants

This study used cross-sectional data from the National Survey on Drug Use and Health (NSDUH), an annual SAMHSA-sponsored survey that measures prevalence and correlates of drug use in a nationwide community sample (https://www.samhsa.gov/data/data-we-collect/nsduh-national-survey-drug-use-and-health). Participants were non-institutionalized individuals aged 12 or older residing in the community. Prospective participants were mailed a lead letter with introductory information and then approached by a door-to-door field interviewer to complete the survey. People residing in institutions (e.g., jails, prisons, nursing homes, hospitals) or experiencing unsheltered homelessness were excluded. No exclusions were made for history of incarceration. Participants received $30 for participating.

As in other research [20], these analyses included pooled data from the 2008–2019 NSDUH surveys and limited the sample to participants aged ≥18. For this study, we also restricted our analyses to male participants. We excluded participants with missing data for recent suicide attempts, arrests, major depression, or substance use.

### Measures

The binary outcome was one or more vs. zero self-reported non-fatal suicide attempts over the year prior to survey completion. We use the term “suicide attempts” as a shorthand for these self-reported non-fatal suicide attempts throughout the rest of the article. The NSDUH phrased their question about suicide attempts: “during the past 12 months, did you try to kill yourself?” No additional information was available regarding the precise timing, setting (while incarcerated or in the community), severity, method, or number of attempts within the 12-month window.

The three main independent variables of interest were any vs. no self-reported arrests, major depression, and substance use disorders over the year prior to survey completion. Arrest status was determined by the question: “not counting minor traffic violations, have you been arrested and booked during the past 12 months?” Men who responded yes to this question had been arrested in the past year and were residing in the community when they completed the survey, indicating that they were not convicted of new major crimes and sentenced to lengthy prison terms. Most of these men were likely arrested on relatively minor charges and either released, convicted of minor crimes and served short jail sentences, assigned to community correctional supervision or otherwise diverted from incarceration, or found not guilty at trial.

Major depression and substance use disorder diagnoses were established from questions based on diagnostic criteria in the *Diagnostic and Statistical Manual of Mental Disorders, 4th Edition (DSM-IV)*. The major depression diagnosis was based on two conditions that both had to be met: [1] the participant reported experiencing at least five out of the nine DSM-IV criteria in their lifetime, with at least one being depressed mood or anhedonia, and [2] affirmative response to the question, “in the past 12 months, did you have a period of time when you felt depressed for two weeks or longer while also having some of the other problems we asked about?” The self-reported substance use disorder diagnoses included abuse or dependence of any of the following 10 substance categories: alcohol, marijuana, cocaine, heroin, hallucinogens, inhalants, pain relievers, tranquilizers, stimulants, and/or sedatives. Substance dependence required positive responses to at least three out of the seven DSM-IV criteria, while substance abuse required two conditions to be met: [1] positive response to at least one out of the four DSM-IV criteria, and [2] the requirements for substance dependence were not met for that substance.

We stratified the sample by race/ethnicity. The racial/ethnic groups that we used were Hispanic/Latinx, Non-Hispanic White, Non-Hispanic Black, and Non-Hispanic people of other races. The “other” races included Non-Hispanic Native American, Non-Hispanic Asian, Non-Hispanic Pacific Islander, and Non-Hispanic people of two or more races/ethnicities. The “other” racial category was quite heterogeneous, even more so than the other racial/ethnic categories, which limited its interpretability. However, the relatively small number of respondents and suicide attempts within these racial/ethnic groups necessitated the use of an aggregate “other” category. For convenience, we will not use the qualifier “Non-Hispanic” when describing the Non-Hispanic racial/ethnic groups henceforth.

Covariates included age (18–25; 26–34; 35–49; ≥50), education, and participation in government assistance programs. Education was coded as high school graduation (yes/no). Government assistance was coded yes/no, and it included supplemental security income, food stamps, cash assistance, and/or non-cash assistance. We decided to use government assistance rather than income as a socioeconomic covariate for these reasons: [1] there is more missing data on the income variable in the NSDUH, [2] the risk factors of interest (arrest, major depression, and substance use disorders) are prevalent among individuals with lower socioeconomic status, so receipt of government benefits may be more relevant.

### Statistical analyses

Our initial analyses described the characteristics of the sample, divided into groups based on race/ethnicity. These statistics included prevalence of suicide attempts, arrest, major depression, substance use disorder, and all covariates. Survey weights and sampling characteristics were applied to all analyses.

Next, we used multiple logistic regression to estimate the associations between each independent variable of interest (arrest, major depression, substance use disorder) and suicide attempts, controlling for one another and all covariates. These logistic regression analyses were also disaggregated by race/ethnicity. Odds ratios were converted into risk ratios for ease of interpretation and to avoid overrepresenting associations [32]. Due to multiple comparisons, we highlighted *p*-values that were < 0.01 and < 0.001, and we reported 99% confidence intervals.

Finally, we took these logistic regression models and applied the Stata post-regression command -punaf- to calculate the adjusted population attributable fraction for suicide attempts and each independent variable of interest (arrest, major depression, substance use disorder). The package -punaf- uses post-regression marginal effects to estimate the population attributable fraction by comparing [1] the prevalence of suicide attempts in the baseline sample to [2] the estimated prevalence of suicide attempts in the counterfactual scenarios in which each variable of interest were eliminated from the sample (i.e., set to zero) [33]. This calculation estimates the percentage of suicide attempts that would theoretically be prevented if each independent variable of interest were eliminated from the population. The command -punaf- provides confidence intervals following the recommendations of Greenland and Drescher [34].

All data that we analyzed are publicly available and de-identified, and therefore did not constitute human subjects research according to the institutional review board at the University of Washington. All statistical analyses were performed with Stata software version 13.1 (STATA Corporation, College Station, TX).

## Results

The study sample included 220,261 men. Among them, 138,020 were White, 25,567 were Black, 35,700 were Hispanic, and 20,974 were other races. Within the “other” racial category, 10,979 were Asian or Pacific Islander, 3229 were Native American, and 6766 were multiracial. After survey weights were applied, the sample represented 112 million men, of whom 67.0% were White, 10.7% were Black, 16.1% were Hispanic, and 7.1% were men of other races. See Table 1 for details.

**Table 1:** Sample characteristics

	All	Non-Hispanic			Hispanic
		White	Black	Other	
*Sample size*					
Unweighted	220,261	138,020	25,567	20,974	35,700
Weighted	112 million	75 million	12 million	8 million	18 million
*Characteristic*	*Weighted Prevalence (%)*				
Suicide attempt	0.4	0.4	0.6	0.5	0.5
Arrest	3.4	2.7	7.2	2.4	4.0
Major depression	5.1	5.6	3.9	4.3	4.2
Substance use disorder	11.1	11.0	11.9	8.6	11.7
Age					
18–25	14.8	12.8	18.2	17.7	20.0
26–34	16.3	14.3	18.0	19.7	22.6
35–49	26.3	24.8	26.8	28.6	31.0
≥ 50	42.6	48.2	37.1	34.1	26.5
Graduated high school	85.9	90.1	81.3	91.1	68.3
Received government benefits	15.4	12.0	28.8	14.4	21.3

The prevalence of suicide attempt(s) in the past year was 0.4% among all men. It differed across race/ethnic groups (p < 0.001): 0.4% among White men, 0.6% among Black men, 0.5% among Hispanic men, and 0.5% among men of other races/ethnicities.

Among all men in the sample, the prevalences of arrest, major depression, and substance use disorder in the past year were 3.4, 5.1, and 11.1%, respectively. All three characteristics differed by race/ethnicity (*p* < 0.001 for each). The prevalence of arrest was 2.7% for White men, 7.2% for Black men, 4.0% for Hispanic men, and 2.4% for men of other races/ethnicities. The prevalence of major depression was 5.6% for White men, 3.9% for Black men, 4.2% for Hispanic men, and 4.3% for men of other races/ethnicities. The prevalence of substance use disorder was 11.0% for White men, 11.9% for Black men, 11.7% for Hispanic men, and 8.6% for men of other races/ethnicities.

Age, education, and government benefits each varied by race/ethnicity (*p* < 0.001 for each). White men were the oldest, Hispanic men were the youngest, and Black men and those of other races/ethnicities were in between. High school graduation or higher education was most prevalent White men (90.1%) and men of other races/ethnicities (91.1%), less prevalent for Black men (81.3%), and least prevalent in Hispanic men (68.3%). Receipt of government benefits was most prevalent among Black men (28.8%), least prevalent among White men (12.0%) and men of other races/ethnicities (14.4%), and in the middle for Hispanic men (21.3%). See Table 1 for the detailed distribution of covariates by race/ethnicity.

### Logistic regression results

Men reporting recent arrest were twice as likely to report a recent suicide attempt as those who did not report recent arrest (RR = 2.1, 99% CI 1.6–2.8, *p* < 0.001). Comparatively, men reporting recent major depression were nearly 11 times more likely to report a recent suicide attempt than those without recent major depression (RR = 10.7, 99% CI 8.8–13.0, *p* < 0.001), and men reporting a recent substance use disorder were 2.5 times as likely to report a recent suicide attempt as men without a recent substance use disorder (RR = 2.5, 99% CI 2.0–3.1, *p* < 0.001).

Disaggregated by race/ethnicity, the association between arrest and suicide attempts was statistically significant among White men (RR = 2.4, 99% CI 1.6–3.4, p < 0.001) and Hispanic men (RR = 2.1, 99% CI 1.1–4.1, *p* = 0.004). It was not significant among men of other racial/ethnic groups: Black men (RR = 1.8, 99% CI 0.9–3.3, *p* = 0.017) and men of other races/ethnicities (RR = 1.3, 99% CI 0.5–3.5, *p* = 0.528). Refer to Table 2 for disaggregation of the major depression and substance use disorder results by race/ethnicity.

**Table 2:** Associations between arrest, major depression, substance use disorders, and suicide

	All	Non-Hispanic			Hispanic
		White	Black	Other	
*Logistic Regression*	*Relative Risk of Suicide Attempt (99% CI)*				
Arrest	2.1** (1.6, 2.8)	2.4** (1.6, 3.4)	1.8 (0.9, 3.3)	1.3 (0.5, 3.5)	2.1* (1.1, 4.1)
Major depression	10.7** (8.8, 13.0)	11.0** (8.2, 14.8)	7.5** (4.2,13.5)	12.7** (6.5, 24.5)	11.0** (6.6, 18.1)
Substance use disorder	2.5** (2.0, 3.1)	2.4** (1.8, 3.2)	3.3** (1.9, 5.7)	2.6* (1.1, 6.2)	2.1* (1.2, 3.8)
*Population Attributable Risk Percent*	*Percentage of Suicide Attempts Attributed to Each Characteristic (99% CI)*				
Arrest	8.9** (5.1, 12.6)	9.5** (4.5, 14.3)	10.2 (−3.0, 21.6)	2.0 (−7.0, 10.3)	8.1 (−0.5, 15.9)
Major depression	40.3** (35.0, 45.1)	44.8** (36.9, 51.7)	27.2** (14.7, 37.8)	41.7** (20.9, 57.0)	35.9** (23.7, 46.1)
Substance use disorder	24.1** (17.6, 30.2)	23.9** (15.7, 31.3)	30.9** (13.7, 44.7)	21.3 (−4.0, 40.5)	19.4* (2.2, 33.6)
All three together	54.6** (49.4, 59.4)	57.9** (51.1, 63.7)	50.2** (36.8, 60.7)	51.3** (28.4, 66.9)	49.2** (39.1, 57.6)

* 0.001 ≤ *p* < 0.01; ** *p* < 0.001

### Population Attributable Risk Percent (PAR%) Results

Among the full weighted sample of men, recent arrest, major depression, and substance use disorder each accounted for a significant fraction of recent suicide attempts (*p* < 0.001). Based on the fully adjusted PAR%, arrest accounted for 8.9% of suicide attempts (99% CI 5.1–12.6%), major depression for 40.3% (99% CI 35.0–45.1%), and substance use disorders for 24.1% (99% CI 17.6–30.2%). When taken together, these three variables accounted for 54.6% of recent suicide attempts (99% CI 49.4–59.4%).

When disaggregated by race, arrest accounted for a significant percentage of suicide attempts among White men (9.5, 99% CI 4.5–14.3%, *p* < 0.001). Although the PAR% estimates were similar, arrest did not account for a significant percentage of suicide attempts among Black men (10.2, 99% CI − 3.0 to 21.6%, *p* = 0.043), Hispanic men (8.1, 99% CI − 0.5 to 15.9%, *p* = 0.016), or men of other races/ethnicities (2.0, 99% CI − 7.0 to 10.3%, *p* = 0.550). See Table 2 for disaggregation of PAR% results for major depression and substance use disorders.

## Discussion

This study found that 8.9% of recent suicide attempts among men were attributable to recent arrest, compared to 40.3% attributable to recent major depression and 24.1% attributable to substance use disorders. When disaggregated by race/ethnicity, recent arrest accounted for 9.5% of all recent suicide attempts among White men. Estimates were similar but not statistically significant for Black and Hispanic men, and much lower for the heterogeneous category of men of other races/ethnicities.

The finding that recent arrest accounted for 8.9% of all recent suicide attempts among U.S. men, or approximately one in eleven, demonstrates the public health significance of arrest. Roughly 50,000 of the adult male suicide attempts per year in America may be related to arrest. The use of survey weights and controlling for other important suicide risk factors including depression, substance use disorders, race/ethnicity, age, and some socioeconomic characteristics indicates that this may be an independent effect. This result is even more surprising given the relatively low prevalence of arrest (3.4%) in this sample. Unlike many other suicide risk factors, criminal arrest is a discrete and identifiable event, which creates an opportunity for intervention. Policies and services to reduce arrest, mitigate the stress and trauma of arrest through mental health training for law enforcement officers, and access to mental health treatment following arrest could all reduce the risk of suicide in this vulnerable population. Understanding the nature of the association between criminal arrest and suicidality is likely to be critical to developing effective policies and interventions.

Prior studies on suicide risk within the criminal legal system inform how we interpret our results. In order to participate in the NSDUH, people who were arrested in the past year had been booked, detained, processed through the legal system (e.g., charges dropped, freed on bail, gone through court, served short sentences, etc.), and released in time to complete the survey. Those who were arrested and remained incarcerated could not participate and would be missing from the study. Therefore, those with the most severe criminal involvement are likely to be excluded. Many of those included in the study would have undergone pre-trial detainment in jail, which is known to be a period of exceptionally high risk for suicide [14, 15]. In addition to the stress and trauma of the criminal arrest itself (and the unstable life circumstances that often precede criminal arrest), these individuals also experienced the stress of pre-trial detainment, including poor jail conditions, legal uncertainty, disruption of life and work, and the stigma of criminal legal involvement [35]. For most, arrest is the point of entry into the criminal legal system, a precipice from which people look upon the future with fear and apprehension. Subsequent release from detention likely comes with its own stresses as people try to put their lives back together, perhaps with the additional pressure of community correctional supervision, although reentry following jail detention has not been studied as a suicide risk factor as well as reentry following prolonged incarceration [19].

Disaggregating our results by race/ethnicity found similar percentages of suicide attempts attributable to arrest in White, Black, and Hispanic men (8–10%), but the estimates for Black and Hispanic men were not statistically significant. The similar results across racial/ethnic groups may be surprising in light of prior research demonstrating (a) the significant mental health consequences of criminal legal involvement for Black men [31, 36, 37], and (b) the history of anti-black racism that underpins mass incarceration and its effects on black communities [27, 38]. In fact, other researchers have also found similar associations between criminal legal involvement and health outcomes across racial/ethnic groups [19, 39, 40]. The concept of “disadvantage saturation” has been invoked in sociological research to explain the lack of variation across race/ethnicity, proposing that the health impacts of additional stresses attenuate once a certain threshold is reached [19, 41, 42]. From a life course perspective, many people who become involved in the criminal legal system as adults may have already reached their threshold of “disadvantage saturation” regardless of race/ethnicity.

Despite the overall similarities across White, Black, and Hispanic men, disaggregation yielded important differences that we would not have noticed had we lumped all men together in a single analysis. Prevalence of arrest and suicide attempts were highest among Black men, lowest among White men, and in between for Hispanic men. However, the association between arrest and suicide was strongest among White men, weakest among Black men, and, again, in between for Hispanic men. These results could speak to some degree of differential “disadvantage saturation” across races/ethnicities. It may also suggest unique strategies are needed to reduce arrest-associated suicide attempts in different racial/ethnic groups. For instance, we anticipate that programs and policies to reduce arrest among Black men, such as less aggressive and discriminatory policing and greater public investment in black communities [43, 44], might have specific benefit, given the high prevalence of arrest in this demographic. Programs and policies to mitigate the association between arrest and suicide, such as enhanced police training around response to behavioral crises, may have a greater impact for White men. These arguments, however, are speculative, and should be taken with appropriate caution.

Despite finding no evidence of significant associations between arrest and suicide in Black, Hispanic, Native American, Asian, Pacific Islander, and multiracial men, we caution against concluding that arrest is not harmful in these racial/ethnic groups. Additional research is needed, and with larger sample sizes, to establish with greater detail and precision the mental health consequences and correlates of arrest.

Our results should be interpreted within the context of several methodological limitations. First, we could not establish the timing of recent suicide attempts relative to recent arrests, major depressive episodes, and substance use disorders. Therefore, we cannot be sure of the direction of causality for any of the three main risk factors analyzed. Second, we were limited by relatively small sample sizes among Native American, Asian, Pacific Islander, and multiracial men, which makes it hard to draw definitive conclusions about suicide in these racial/ethnic groups. Third, all racial/ethnic groups are fairly broad and have substantial ethnic variation within them that could mask important differences. Fourth, our analyses are vulnerable to unmeasured and residual confounding, especially by psychiatric diagnoses other than major depression and socioeconomic variables. Despite these limitations, we believe that our results provide important preliminary results on the link between arrest and suicide among men, and within the context of other important suicide risk factors.

## Conclusions

Arrest, major depression, and substance use disorders each accounted for a significant proportion of suicide attempts among U.S. men. Roughly 50,000 of the adult male suicide attempts per year in America may be related to arrest. Our results were similar for Non-Hispanic White, Non-Hispanic Black, and Hispanic men, although only significant for White men, and there were differences in prevalence of arrest and suicide attempts. As a means to reduce public health disparities, suicide prevention programs should account for arrest and racial differences. The post-arrest period may be an important time to provide mental health support.

## Data Availability

All data used in this study are available through the Inter-University Consortium for Political and Social Research (ICPSR) at https://icpsr.umich.edu/web/ICPSR/series/64. It is also available through the Substance Abuse and Mental Health Data Archive (SAMHDA) at https://datafiles.samhsa.gov/study-series/national-survey-drug-use-and-health-nsduh-nid13517.

## Abbreviations

PAR%: Population attributable risk percent
NSDUH: National Survey on Drug Use and Health
SAMHSA: Substance Abuse and Mental Health Services Administration
DSM-IV: Diagnostic and Statistical Manual of Mental Disorders, 4th Edition
RR: Risk ratio

## References

[1] Nock MK, Hwang I, Sampson NA, Kessler RC (2010). Mental disorders, comorbidity and suicidal behavior: results from the National Comorbidity Survey Replication. Mol Psychiatry.

[2] Yeh H-H, Westphal J, Hu Y, Peterson EL, Williams LK, Prabhakar D, Frank C, Autio K, Elsiss F, Simon GE, Beck A, Lynch FL, Rossom RC, Lu CY, Owen-Smith AA, Waitzfelder BE, Ahmedani BK (2019). Diagnosed mental health conditions and risk of suicide mortality. Psychiatr Serv.

[3] Borges G, Walters EE, Kessler RC (2000). Associations of substance use, abuse, and dependence with subsequent suicidal behavior. Am J Epidemiol.

[4] Bohnert KM, Ilgen MA, Louzon S, McCarthy JF, Katz IR (2017). Substance use disorders and the risk of suicide mortality among men and women in the US veterans health administration. Addiction..

[5] Li Z, Page A, Martin G, Taylor R (2011). Attributable risk of psychiatric and socioeconomic factors for suicide from individual-level, population-based studies: a systematic review. Soc Sci Med.

[6] Krysinska K, Martin G (2009). The struggle to prevent and evaluate: application of population attributable risk and preventive fraction to suicide prevention research. Suicide Life Threat Behav.

[7] Bolton JM, Robinson J (2010). Population-attributable fractions of Axis I and Axis II mental disorders for suicide attempts: findings from a representative sample of the adult, noninstitutionalized US population. Am J Public Health.

[8] Bernal M, Haro JM, Bernert S, Brugha T, de Graaf R, Bruffaerts R, Lépine JP, de Girolamo G, Vilagut G, Gasquet I, Torres JV, Kovess V, Heider D, Neeleman J, Kessler R, Alonso J, ESEMED/MHEDEA Investigators (2007). Risk factors for suicidality in Europe: results from the ESEMED study. J Affect Disord.

[9] Cheung YB, Law CK, Chan B (2006). Suicidal ideation and suicide attempts in a population-based study of Chinese people: risk attributable to hopelessness, depression, and social factors. J Affect Disord.

[10] Ramsawh HJ, Fullerton CS, Herberman Mash HB (2014). Risk for suicidal behaviors associated with PTSD, depression, and their comorbidity in the U.S. Army. J Affect Disord.

[11] Chan SSM, Chiu HFK, Chen EYH (2009). Population-attributable risk of suicide conferred by Axis I psychiatric diagnoses in a Hong Kong Chinese population. Psychiatr Serv.

[12] Pirkis J, Burgess P, Dunt D (2000). Suicidal ideation and suicide attempts among Australian adults. Crisis..

[13] Bruffaerts R, Kessler RC, Demyttenaere K, Bonnewyn A, Nock MK (2015). Examination of the population attributable risk of different risk factor domains for suicidal thoughts and behaviors. J Affect Disord.

[14] Dying Inside: The hidden crisis in America’s jails. Reuters. Available at https://reuters.com/investigations/section/usa-jails. Accessed 20 Sept 2021.

[15] Noonan ME, Rohloff H, Ginder S. Mortality in local jails and state prisons, 2000-2013 – statistical tables. U.S. Department of Justice, Office of Justice Programs, Bureau of Justice Statistics Available at https://bjs.ojp.gov/content/pub/pdf/mljsp0013st.pdf. Accessed Sept 20, 2021.

[16] Binswanger IA, Stern MF, Deyo RA, Heagerty PJ, Cheadle A, Elmore JG, Koepsell TD (2007). Release from prison – a high risk of death for former inmates. N Engl J Med.

[17] Zlodre J, Fazel S (2012). All-cause and external mortality in released prisoners: systematic review and meta-analysis. Am J Public Health.

[18] Pratt D, Piper M, Appleby L, Webb R, Shaw J (2006). Suicide in recently released prisoners: a population-based cohort study. Lancet.

[19] Sugie NF, Turney K (2017). Beyond incarceration: criminal justice contact and mental health. Am Sociol Rev.

[20] Bryson WC, Piel J, Thielke S (2020). Associations between parole, probation, arrest, and self-reported suicide attempts. Community Ment Health J.

[21] DeVylder JE, Frey JJ, Cogburn CD (2017). Elevated prevalence of suicide attempts among victims of police violence in the USA. J Urban Health.

[22] Cook TB (2013). Recent criminal offending and suicide attempts: a national sample. Soc Psychiatry Psychiatr Epidemiol.

[23] Cooper H, Moore L, Gruskin S, Krieger N (2004). Characterizing perceived police violence: implications for public health. Am J Public Health.

[24] Edwards ER, Gromatsky M, DRG S, et al. Arrest history and psychopathology among veterans at risk for suicide. Psychol Serv. 2020; epub ahead of print. Available at. 10.1037/ser0000454.10.1037/ser000045433119341

[25] Lamb HR, Weinberger LE, Gross BH (2004). Mentally ill persons in the criminal justice system: some perspectives. Psychiatry Q.

[26] Tiu RT, Trout ZM, Hernandez EM (2017). A behavioral and cognitive neuroscience perspective on impulsivity, suicidal, and non-suicidal self-injury: meta-analysis and recommendations for future research. Neurosci Biobehav Rev.

[27] Alexander M (2010). The new Jim crow: mass incarceration in the age of colorblindness.

[28] O’Reilly KB. AMA: racism is a threat to public health. American Medical Association https://www.ama-assn.org/delivering-care/health-equity/ama-racism-threat-public-health. Accessed 21 Jan 2021.

[29] APA Condemns police brutality, calls for dialogue to ease civil unrest. American Psychiatric Association. https://www.psychiatry.org/newsroom/news-releases/apa-condemns-police-brutality-calls-for-dialogue-to-ease-civil-unrest. Accessed 21 Jan 2021.

[30] NACCHO Statement of policy 16-05. Health equity: mass incarceration and structural racism. National Association of County & City Health Officials https://www.naccho.org/uploads/downloadable-resources/16-05-Health-Equity-Mass-Incarceration-and-Structural-Racism.pdf. Accessed 21 Jan 2021.

[31] APA position statement on police brutality and black males. American Psychiatric Association. http://www.psychiatry.org/File Library/About-APA/Organization-Documents-Policies/Policies/Position-Police-Brutality-and-Black-Males.pdf. Accessed 21 Jan 2021.

[32] Wang Z (2013). Converting odds ratio to relative risk in cohort studies with partial data information. J Stat Softw.

[33] Newsom RB (2013). Attributable and unattributable risks and fractions and other scenario comparisons. Stata J.

[34] Greenland S, Drescher K (1993). Maximum likelihood estimation of the attributable fraction from logistic models. Biometrics..

[35] Patton DE, Vars FE (2020). Jail suicide by design. UCLA Law Rev Dis.

[36] Tonry M, Melewski M (2008). The malign effects of drug and crime control policies on black Americans. Crime Justice.

[37] Bor J, Venkataramani AS, Williams DR, Tsai AC (2018). Police killings and their spillover effects on the mental health of black Americans: a population-based, quasi-experimental study. Lancet.

[38] Haney Lopez IF (2010). Post-racial racism: racial stratification and mass incarceration in the age of Obama. Cal L Rev.

[39] Massoglia M (2008). Incarceration, health, and racial disparities in health. Law Soc Rev.

[40] Turney K, Wildeman C, Schnittker J (2012). As fathers and felons: explaining the effects of current and recent incarceration on major depression. J Health Soc Behav.

[41] Hannon L (2003). Poverty, delinquency, and educational attainment: cumulative disadvantage or disadvantage saturation?. Sociol Inq.

[42] Krivo LJ, Peterson RD (2000). The structural context of homicide: accounting for racial differences in process. Am Sociol Rev.

[43] Hall T, Wooten NR, Lundgren LM (2015). Postincarceration policies and prisoner reentry: implications for policies and programs aimed at reducing recidivism and poverty. J Poverty.

[44] Reducing racial disparity in the criminal justice system: a manual for practitioners and policymakers. The Sentencing Project. https://www.sentencingproject.org/wp-content/uploads/2016/01/Reducing-Racial-Disparity-in-the-Criminal-Justice-System-A-Manual-for-Practitioners-and-Policymakers.pdf. Accessed 21 Jan 2021.

